# *ACOX1*, regulated by C/EBPα and miR-25-3p, promotes bovine preadipocyte adipogenesis

**DOI:** 10.1530/JME-20-0250

**Published:** 2021-01-22

**Authors:** Feng Zhang, Qi Xiong, Hu Tao, Yang Liu, Nian Zhang, Xiao-Feng Li, Xiao-Jun Suo, Qian-Ping Yang, Ming-Xin Chen

**Affiliations:** 1 Hubei Key Laboratory of Animal Embryo Engineering and Molecular Breeding, Institute of Animal Husbandry and Veterinary, Hubei Academy of Agricultural Sciences, Wuhan, China

**Keywords:** bovine, ACOX1, CEBPα, miR-25-3p, adipogenesis

## Abstract

Acyl-coenzyme A oxidase 1 (ACOX1) is the first and rate-limiting enzyme in peroxisomal fatty acid β-oxidation of fatty acids. Previous studies have reported that *ACOX1* was correlated with the meat quality of livestock, while the role of *ACOX1* in intramuscular adipogenesis of beef cattle and its transcriptional and post-transcriptional regulatory mechanisms remain unclear. In the present study, gain-of-function and loss-of-function assays demonstrated that *ACOX1* positively regulated the adipogenesis of bovine intramuscular preadipocytes. The C/EBPα-binding sites in the bovine *ACOX1* promoter region at −1142 to −1129 bp, −831 to −826 bp, and −303 to −298 bp were identified by promoter deletion analysis and site-directed mutagenesis. Electrophoretic mobility shift assays (EMSA) and chromatin immunoprecipitation (ChIP) further showed that these three regions are C/EBPα-binding sites, both* in vitro* and* in vivo*, indicating that C/EBPα directly interacts with the bovine *ACOX1* promoter and inhibits its transcription. Furthermore, the results from bioinformatics analysis, dual luciferase assay, site-directed mutagenesis, qRT-PCR, and Western blotting demonstrated that miR-25-3p directly targeted the *ACOX1* 3’UTR (3’UTR). Taken together, our findings suggest that *ACOX1*, regulated by transcription factor C/EBPα and miR-25-3p, promotes adipogenesis of bovine intramuscular preadipocytes via regulating peroxisomal fatty acid β-oxidation.

## Introduction

Acyl-coenzyme A oxidase (ACOX) and mitochondrial acyl-CoA dehydrogenase belong to the same flavoenzyme superfamily and have evolved from the same progenitor (Kunau *et al.* 1995). ACOX1 is the first and rate-limiting enzyme in peroxisomal fatty acid β-oxidation of fatty acids of all eukaryotes: acyl-CoAs longer than C8 were desaturated to 2-*trans*-enoyl-CoAs, donating electrons directly to molecular oxygen, thus generating H_2_O_2_ and energy, lost as heat (Li *et al.* 2000, Morais *et al.* 2007). ACOX1 is a highly conserved enzyme with a unique expression pattern, its mRNA and protein expression were most abundant in liver, followed by kidney, brain and adipose tissue (Nohammer *et al.* 2000).

Previous studies have reported that ACOX1 plays an important role in lipid metabolism. Inhibition of ACOX1 was a novel and effective approach for the treatment of high-fat diet or obesity induced metabolic diseases by improving mitochondrial lipid and reactive oxygen species (ROS) metabolism (Zeng *et al.* 2017). The down-expression of PPARα and ACOX1 in liver of rats with alcoholic fatty liver disease suppressed fatty acid metabolism and leaded to triglyceride (TG) deposition in the liver (Tong *et al.* 2016). siRNA knockdown of *ACOX1* strongly increased the levels of very long chain fatty acids (VLCFA) and neutral lipids (Baarine *et al.* 2012). Besides, several studies have found that ACOX1 was correlated with the meat quality of livestock. Phenotype analysis of 334 Large White × Meishan F_2_ pigs showed that *Pst* I variants of *ACOX1* gene significantly affected the meat color value and meat marble score of both longissimus dorsi and biceps femoris (Zuo *et al.* 2007). Porcine *ACOX1* gene was most closely linked to significant quantitative trait loci (QTL) affecting average daily gain, birth weight, backfat thickness, and fatty acid composition (Casas Carrillo *et al.* 1997, Clop *et al.* 2003, Yue *et al.* 2003). A SNP in exon 13 of bovine* ACOX1* gene resulted in significant differences in backfat thickness and meat marble score among genotypes (Jiao *et al.* 2011). The g.224G > A SNP located in *ACOX1* coding regions was significantly associated with meat quantity grade at slaughter and backfat thickness tended to be greater in Korean cattle (Lee *et al.* 2010). However, to our knowledge, the role of *ACOX1* in intramuscular adipogenesis of beef cattle has not been reported, and its transcriptional and post-transcriptional regulatory mechanisms are not clear.

Thus, in this study, we first investigated the role of *ACOX1* in adipogenesis by gain-of-function and loss-of-function assays. Then, the promoter of bovine *ACOX1* was identified, and the binding sites of the transcription factor CCAAT enhancer-binding proteins alpha (C/EBPα), which is a critical transcription factors in fat deposition and adipocyte differentiation, were predicted and verified using bioinformatics software and experiments; And, the transcriptional activity of *ACOX1* was depressed by C/EBPα. Finally, the targeted site of miR-25-3p in bovine *ACOX1* 3’ UTR was predicted and verified, and the post-transcriptional activity of *ACOX1* was depressed by miR-25-3p.

## Materials and methods

### Bovine intramuscular preadipocytes isolation

Dabieshan yellow cattles (24–30 months old, male) were provided by Hubei Hegen Agricultural Technology Ltd and harvested at a local abattoir using standard procedures. Bovine intramuscular preadipocytes were isolated from longissimus dorsi muscle, the method was as follows. The longissimus dorsi muscle was washed five times with PBS containing 5% penicillin/streptomycin and transported to laboratory in PBS. The following procedures were conducted in a sterile field. Adipose tissues were separated from muscle bundles and finely chopped into 1-mm^3^ pieces with scissors in PBS and then incubated with 0.1% collagenase type I (Sigma) for 1 h at 37°C with mixing every 10 min. After enzymatic digestion, the released fat stromal cells were suspended in DMEM (Gibco) supplemented with 15% fetal bovine serum (FBS; Gibco), and the suspension was filtered through a 100 µm filter (Corning Incorporated). Then, the cells were collected by centrifugation at 650 **g** for 5 min. The cells were added to fresh DMEM supplemented with 15% FBS and 1% penicillin/streptomycin. The cells were then plated in nunclon flasks and cultured in an atmosphere of 5% CO_2_ at 37°C. After 12 h, the non-adherent cells were removed. When cells achieved 80% to 90% confluence, they were passaged by trypsinisation.

### Differentiation induction and oil red O staining

For evaluating the effect of *ACOX1* on adipogenic differentiation of bovine intramuscular preadipocytes, bovine intramuscular preadipocytes were seeded in 6-well plates the day before transfection. pCDNA-ACOX1, pCDNA-3.1(+), Si-ACOX1 and negative control (NC) were transfected into confluent (~80%) cells, respectively. After 24 h, adipogenic differentiation of bovine intramuscular preadipocytes were induced in a medium comprising DMEM supplemented with 10% FBS, 0.5 mM 3-isobutyl-1-methylxanthine, 1 μΜ dexamethasone, and 10 μg/mL of insulin (both from Sigma) for 2 days (from day 0 to day 2). The medium was then replaced with 10 μg/mL insulin in 10% FBS supplemented medium for an additional 2 days (from day 2 to day 4). Lastly, the medium was replaced with 10% FBS supplemented medium (from day 4 to day 8).

On day 8, medium was discarded and cells were washed twice with PBS, fixed in 4% paraformaldehyde for 0.5-1 h and washed again with PBS. The cells were then stained with Oil Red O (0.5 g Oil Red O; Sigma) in 100 mL isopropanol diluted with water (60:40) for 1 h. After being stained, the cells were washed twice in PBS and then photographed. The lipid accumulation of stained cells was qualified by measuring its absorbance at the wavelength of 550 nm (OD_550_).

### Triglyceride content, ATP, and ROS assays

For detecting the concentrations of triglyceride, ATP, and ROS, bovine intramuscular preadipocytes were seeded in 24-well plates the day before transfection. pCDNA-ACOX1, pCDNA-3.1(+), Si-ACOX1 and NC were transfected into confluent (~80%) cells, respectively. After 24–48 h, the concentrations of triglyceride and ATP in the lysates of cells were measured with commercial kits (Applygen (Beijing, China) and Beyotime (Shanghai, China), respectively) following the manufacturer’s instructions, and normalized to the protein content (µ mol/mg protein) using the BCA assay kit (Thermo Scientific). ROS were measured using the reactive oxygen species assay kit (Beyotime) following the manufacturer’s protocol.

### RNA isolation and qRT-PCR

For quantifying the mRNA expression of genes, cells were seeded in six-well plates. After 48 h of the transfection, cells were harvested and total RNA was isolated using a HP Total RNA Kit (Omega, Norcross, GA, USA) according to the manufacturer’s protocol. The cDNA was synthesized using a PrimeScript ™ RT reagent Kit with gDNA Eraser (Takara) according to the manufacturer’s protocol. The qRT-PCR was performed in triplicate with iQSYBR green Supermix (Bio-Rad) in a LightCycler480 Realtime PCR machine (Roche). The mRNA levels of target genes were reported relative to those of the house keeping gene β*-actin* by using the 2^−∆∆Ct^ method. The qRT-PCR primers are listed in Supplementary Table 1 (see section on supplementary materials given at the end of this article).

### Protein isolation and Western blotting

For detecting the protein expression of genes, cells were seeded in 6-well plates. After 48 h of the transfection, cells were harvested and total protein was isolated using RIPA Lysis Buffer (Beyotime). The cells were washed briefly with cold PBS (4°C), 150 µL RIPA Lysis Buffer (containing 1 mM PMSF) was added, incubated for 1 min at room temperature, and then centrifuged at 12,000 ***g*** for 5 min. The supernatant extract was used for Western blot analysis.

Protein concentrations were determined by BCA assay (Thermo Scientific). Proteins boiled in 5× SDS buffer for 5 min were subjected to 5% SDS-PAGE gels, and then transferred to PVDF membranes (Millipore). The membranes were blocked with skim milk and probed with primary antibodies against ACOX1 (Abcam), C/EBPβ (Cell Signaling Technology), FAS (Cell Signaling technology), ACC (Cell Signaling Technology), HSL (Cell Signaling Technology) and LPL (abclonal, Wuhan, China), respectively. β-actin (Santa Cruz Biotechnology) served as the loading control. The results were visualized with horseradish peroxidase-conjugated secondary antibodies (KPL, Gaithersburg, MD, USA) and enhanced chemiluminescence.

### Plasmid construction, cell culture, transient transfection and analysis

Based on the *Bostaurus ACOX1* gene sequence (accession number: NC_037346.1), five *ACOX1* promoter deletion fragments were amplified from the bovine genome via PCR with the primers listed in Supplementary Table 2. Then, the purified PCR products were digested with *Kpn* I and *Xho* I (Fermentas, Lithuania) and ligated into the pGL3-Basic vector (Promega). The obtained plasmids were designated ACOX1-P (1–5). Binding site mutations were generated with mutagenic primers (Supplementary Table 2) using overlap-extension PCR. Bovine kidney cells (MDBK) were cultured in DMEM supplemented with 10 % FBS under 5% CO_2_ at 37°C. For luciferase reporter assays, MDBK cells were seeded in 48-well plates. After 12–16 h, the plated cells were transfected with a recombinant plasmid using Lipofectamine 2000 (Invitrogen) according to the methods of Deng et al (Deng *et al.* 2016).

The potential target site of miR-25-3p, localized in the 3’UTR of *ACOX1* mRNA, was predicted by TargetScan. The ACOX1-3’UTR was amplified from bovine cDNA and inserted into the PmeI/XhoI sites of the pmirGLO vector (Promega). Point mutation and deletion in the seed region of the predicted miR-25-3p sites within the ACOX1-3’UTR were generated using overlap-extension PCR. The corresponding primers are listed in Supplementary Table 3. The luciferase reporter assays could follow a previously described method (Zhang *et al.* 2018) .

### Electrophoretic mobility shift assays

For electrophoretic mobility shift assays (EMSAs), nuclear proteins (NPs) were extracted from bovine longissimus dorsi muscle by using a Nucleoprotein Extraction Kit (Beyotime). Single-stranded oligonucleotides (Supplementary Table 4) corresponding to the C/EBPα-binding sites in the *ACOX1* promoter were synthesized (Aoke, Wuhan, China) and annealed to obtain double-stranded oligonucleotides. The DNA-binding activity of the C/EBPα protein was detected by using a LightShift Chemiluminescent EMSA Kit (Thermo Scientific) following a previously described method (Deng *et al.* 2016).

### Chromatin immunoprecipitation assay

Chromatin immunoprecipitation (ChIP) assays were performed by using EZ-ChIP Kit-17-371 (Millipore) following a previously described method (Deng *et al.* 2016). Precleared chromatin was incubated with the C/EBPα antibody (Abcam) or normal rabbit IgG (Abcam) antibody overnight at 4°C. Purified DNA from the samples and the input controls were analyzed for the presence of *ACOX1* promoter sequences containing putative C/EBPα response elements using qRT-PCR. The primers used here are listed in Supplementary Table 5.

### Bioinformatics

Transcription factor binding sites were predicted by using AliBaba2.1 (http://www.gene-regulation.com/) (Wei *et al.* 2016) and MatInspector (http://www.genomatix.de/online_help/help_matinspector/matinspector_help.html) (Quandt *et al.* 1995, Cartharius *et al.* 2005). The potential target site of miR-25-3p in *ACOX1* 3’UTR was predicted by TargetScan (http://www.targetscan.org/) (Huang *et al.* 2016, Wang *et al.* 2020).

### Statistical analysis

All the results are presented as the means ± s.d. Student’s *t*-test was used for statistical comparisons. A *P* value of < 0.05 was considered to be statistically significant. **P  < 0.01; *P < 0.05; NS, not significant.

## Results

### *ACOX1* promotes adipogenesis of bovine intramuscular preadipocytes *in vitro*

**Figure 1:**
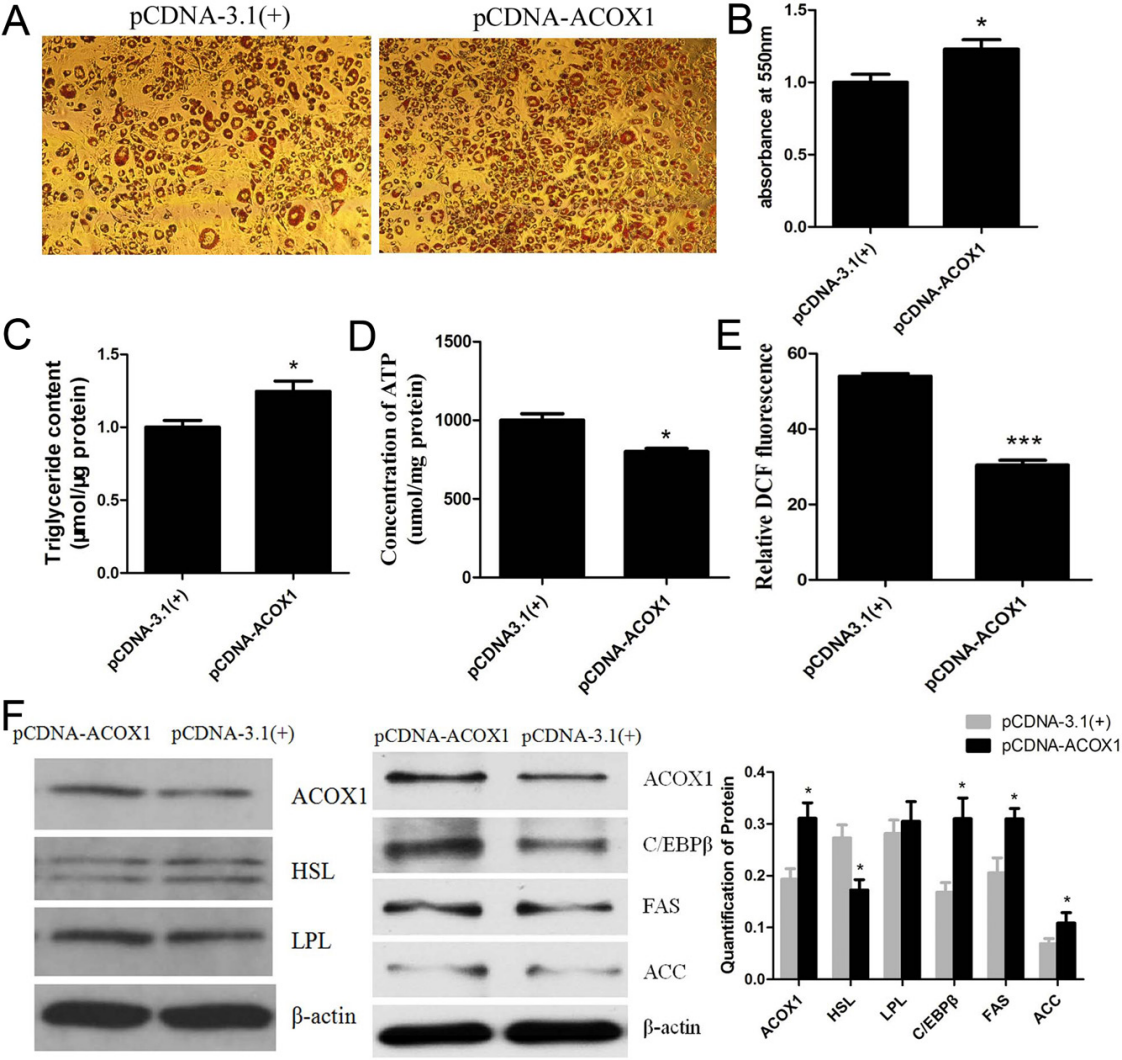
Overexpression of *ACOX1* promotes adipogenesis of bovine intramuscular preadipocytes. The pCDNA-ACOX1 eukaryotic expression plasmid was transfected into bovine intramuscular preadipocytes. Following a 24 h transfection, the cells were induced to undergo adipogenic differentiation, and stained with Oil Red O on day 8 (A) and lipid drops content was measured by OD 550 nm (B). After 24–48 h transfection, the content of triglyceride (C), ATP (D) and Reactive Oxygen Species (ROS) (E) were measured with the commercial kits. The fluorescence of DCF represents the content of ROS. (F) After 48 h transfection, the expression of HSL, LPL, C/EBPβ, FAS and ACC were detected by Western blotting. β-actin served as the loading control. pCDNA-3.1(+) was used as a negative control. Data were presented as means ± s.d. (*n* ≥ 3), *P < 0.05; ***P < 0.001.

To investigate whether *ACOX1* was related to adipogenesis of bovine intramuscular preadipocytes, we isolated bovine intramuscular preadipocytes and performed *ACOX1* gain-of-function and loss-of-function experiments. The pCDNA-ACOX1 eukaryotic expression plasmid was constructed and transfected into bovine intramuscular preadipocytes. Following a 24 h transfection, the cells were induced to undergo adipogenic differentiation, and Oil Red O staining on day 8 showed that over-expression of *ACOX1* significantly promoted lipid accumulation (Fig. 1A and B). In addition, the concentrations of triglyceride, adenosine 5’-triphosphate (ATP), and ROS were detected after a 24–48 h transfection. The results showed that over-expression of *ACOX1* significant increased levels of triglyceride, whereas significant decreased levels of ATP and ROS (Fig. 1C,D and E). Furthermore, *ACOX1* over-expression promoted CCAAT/enhancer binding protein beta (C/EBPβ), fatty acid synthase (FAS), and acetyl-CoA carboxylase (ACC) expressions, whereas inhibited hormone sensitive lipase (HSL) expression, there was no significant effect on the expression of lipoprotein lipase (LPL), measured by Western blotting (Fig. 1F).

**Figure 2:**
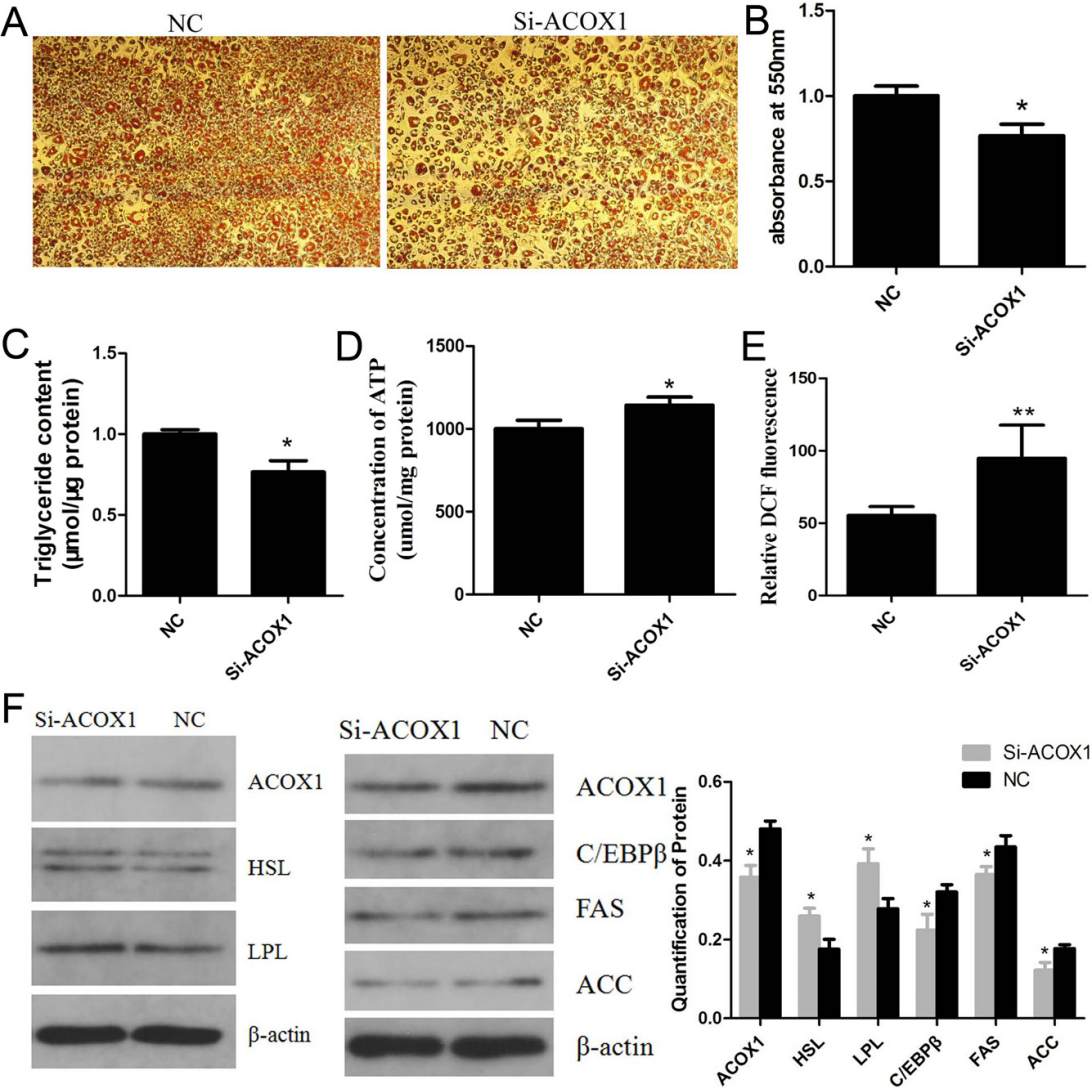
Knockdown of *ACOX1* inhibits adipogenesis of bovine intramuscular preadipocytes. small interference RNA (siRNA) against *ACOX1* was transfected into bovine intramuscular preadipocytes. Following a 24 h transfection, the cells were induced to undergo adipogenic differentiation, and stained with Oil Red O on day 8 (A) and lipid drops content was measured by OD 550 nm (B). After 24–48 h transfection, the content of triglyceride (C), ATP (D) and Reactive Oxygen Species (ROS) (E) were measured with the commercial kits. The fluorescence of DCF represents the content of ROS. (F) After 48 h transfection, the expression of HSL, LPL, C/EBPβ, FAS and ACC were detected by Western blotting. β-actin served as the loading control. NC, negative control. Data were presented as means ± s.d. (*n* ≥ 3), *P < 0.05; **P < 0.01.

Three different small interference RNAs (siRNA) against *ACOX1* were synthesized by RiboBio (Guangzhou, China) and transfected into bovine intramuscular preadipocytes. The efficacy of the siRNA-mediated knockdown was shown in Supplementary Fig. 1. *ACOX1* mRNA expression was significantly decreased by Si-ACOX1-2, which was chosen for subsequent experiments. Oil Red O staining on day 8 showed that Si-ACOX1 significantly diminished the accumulation of lipid droplets (Fig. 2A and B). Triglyceride level was decreased, while ATP and ROS levels were increased by Si-ACOX1 compared with the negative control (NC) siRNA (Fig. 2C, D and E). Moreover, C/EBPβ, FAS, and ACC expressions were suppressed, while HSL and LPL expressions were promoted by Si-ACOX1 (Fig. 2F). Thus, the combined data from gain- and loss-of-function studies consistently demonstrate that *ACOX1* promotes adipogenesis of bovine intramuscular preadipocytes.

### Isolation and transcriptional activity assay of the bovine *ACOX1* promoter

**Figure 3:**
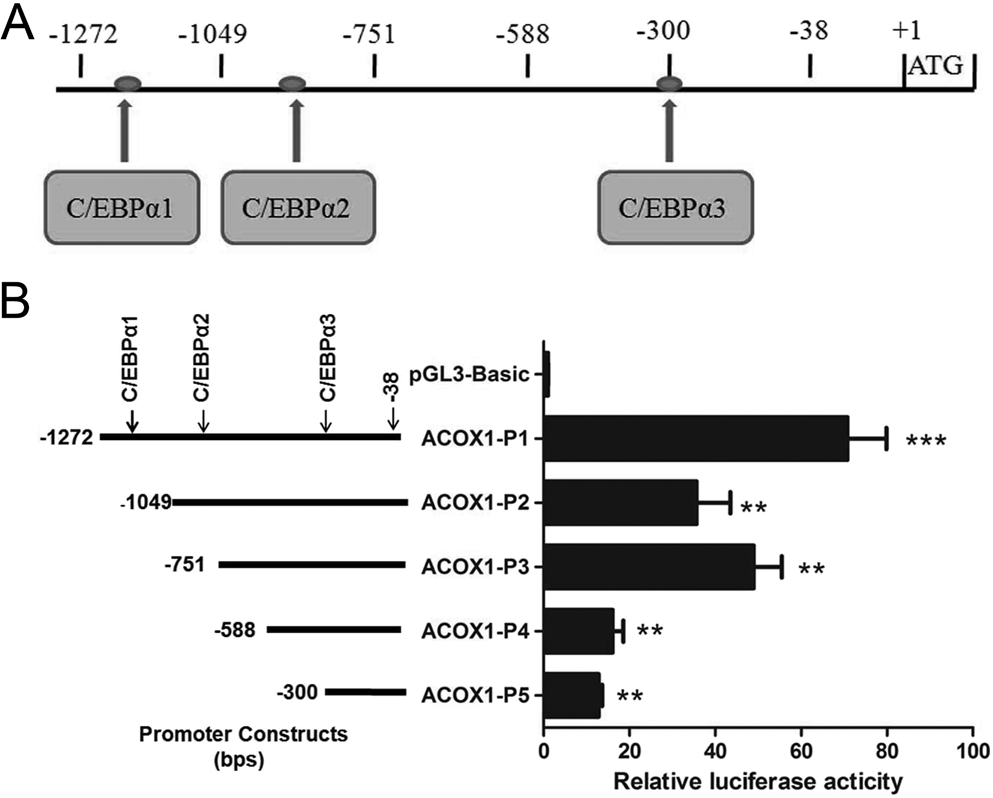
*ACOX1* 5’-deletion constructs and dual-luciferase reporter assay in Bovine kidney cells (MDBK). (A) Schematic diagram of the C/EBPα binding sites (arrow, solid red circle) in the *ACOX1* promoter. The first nucleotide of translation initiation site was assigned as +1, and the nucleotides were numbered relative to it. (B) Five deletion constructs were transfected into MDBK cells, data were expressed as the ratio of relative activity normalized to pRL-TK and then normalized to the activity of pGL3-Basic; they were presented as means ± s.d. (*n* ≥ 3), **P < 0.01; ***P < 0.001.

A 1235 bp fragment of the 5’-flanking region of the bovine *ACOX1* gene was obtained from Dabieshan yellow cattle genomic DNA by PCR. Three putative C/EBPα binding sites were predicted within the 5’-flanking region by AliBaba2.1 and MatInspector (Fig. 3A). To determine whether the isolated 5’-flanking region exhibited promoter activity, this fragment and corresponding fragments with progressive deletions were inserted into a luciferase reporter vector (pGL3-Basic). The plasmids containing the various lengths of the *ACOX1* promoter were then transiently transfected into Bovine kidney cells (MDBK). Analyses of luciferase activity revealed that all the deletion vectors have transcriptional activity compared with pGL3-Basic, and ACOX1-P1 (−1272/−38) was the greatest (Fig. 3B). However, the longer fragment showed lower transcriptional activity, suggesting the presence of one or more *cis*-acting elements between −1049 and −751 bp that can inhibit *ACOX1* expression. Moreover, the shorter fragments in ACOX1-P5 to ACOX1-P3 displayed increased transcriptional activity, indicating that the region from −751 to −300 bp contains the *cis*-acting elements that can induce *ACOX1* expression.

### *ACOX1* transcriptional activity was down-regulated by C/EBPα

**Figure 4:**
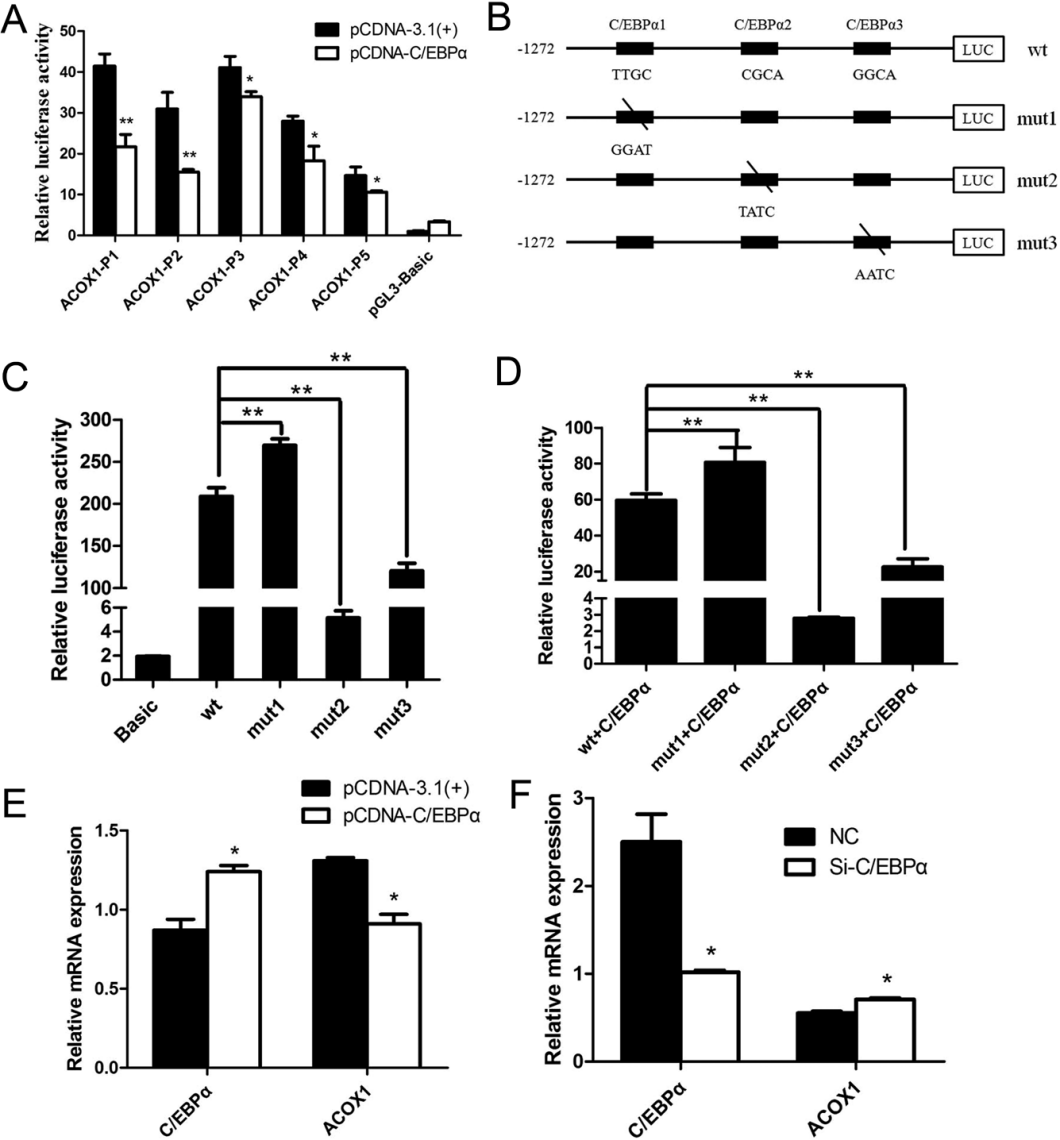
C/EBPα inhibits the transcription of the bovine *ACOX1* gene. (A) Five deletion constructs were co-transfected with pCDNA-C/EBPα into MDBK cells. Over-expression of C/EBPα downregulated *ACOX1* luciferase activity. (B) The schematic diagram of site-directed mutagenesis in the predicted C/EBPα binding sites in the *ACOX1* promoter. (C and D) A series of mutants of three C/EBPα binding sites were constructed and transfected or co-transfected with pCDNA-C/EBPα into MDBK cells. Data were expressed as the ratio of relative activity normalized to pRL-TK. (E) The pCDNA-C/EBPα eukaryotic expression plasmid was transfected into MDBK cells. After 24 h, *C/EBPα* and *ACOX1* expression was determined by qRT-PCR. (F) Small interference RNA (siRNA) against *C/EBPα* was transfected into MDBK cells. After 24 h, *C/EBPα* and *ACOX1* expression was determined by qRT-PCR. NC, negative control. pCDNA-3.1(+) was used as a negative control. Data were presented as means ± s.d. (*n* ≥ 3), *P < 0.05; **P < 0.01.

Five *ACOX1* promoter deletion vectors were each co-transfected with pCDNA-C/EBPα into MDBK cells to determine the effect of C/EBPα on *ACOX1* promoter activity. Co-transfection with pCDNA-C/EBPα significantly suppressed *ACOX1* promoter transcriptional activity for all of the fragments (Fig. 4A). To determine the functional importance of the C/EBPα-binding sites, we individually mutated the C/EBPα binding site at −1142 to −1129 bp, −831 to −826 bp, and −303 to −298 bp by using WT pGL3-ACOX1-P1 as the template (Fig. 4B). A series of mutants of these sites were constructed and transfected or co-transfected with pCDNA-C/EBPα into MDBK cells. Promoter transcriptional activity was significantly increased for mut1, while significantly decreased for mut2 and mut3 compared with the wild construct (Fig. 4C and D). These results suggested that the first binding site is more likely to be the C/EBPα binding site, the second and third sites may also combine with other transcription factors that promote *ACOX1* transcription.

To investigate whether C/EBPα regulates *ACOX1* expression, over-expression and RNA interference experiments of *C/EBPα* were carried out respectively. Three siRNAs against *C/EBPα* were synthesized by RiboBio (Guangzhou, China) and transfected into MDBK cells*. C/EBPα* mRNA expression was significantly decreased by Si-C/EBPα-1 (Supplementary Fig. 2), which was chosen for subsequent experiments. pCDNA-C/EBPα, pCDNA-3.1(+), Si-C/EBPα and NC were transfected into MDBK cells, respectively. After 48 h of the transfection, total RNA was isolated. The over-expression of *C/EBPα* resulted in the significant suppression of *ACOX1* expression, while the knockdown of *C/EBPα* significant increased *ACOX1* expression by qRT-PCR (Fig. 4Eand F). These results suggested that C/EBPα inhibited *ACOX1* expression.

### Transcription factor C/EBPα binds to *ACOX1* promoter both *in vitro* and *in vivo*

**Figure 5:**
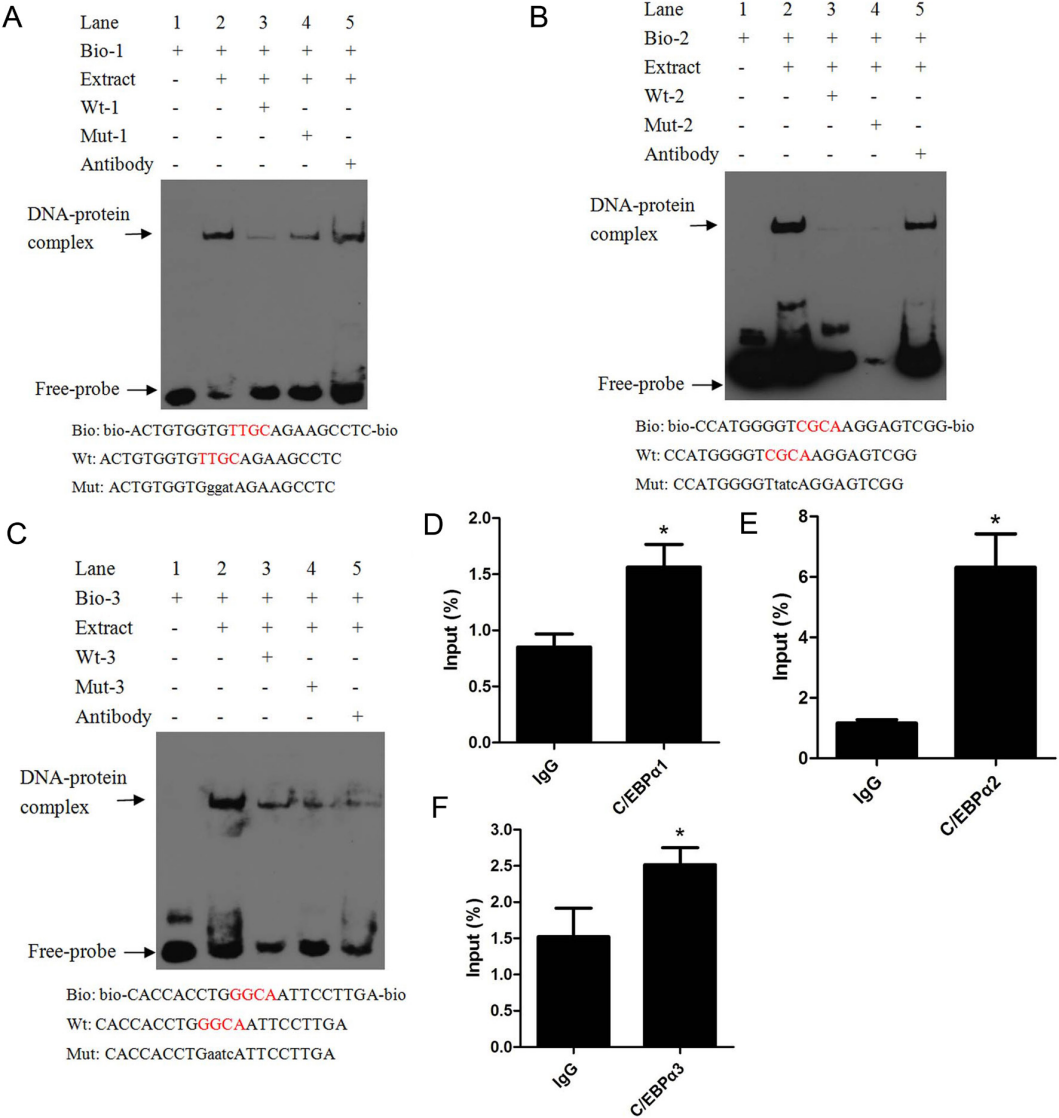
Binding of C/EBPα to *ACOX1* promoter region was analyzed by electrophoretic mobility shift assay (EMSA) and chromatin immunoprecipitation (ChIP). (A, B and C) Probe was incubated with nuclear proteins extract in the absence or presence of a 100-fold excess of various competitor probes (mutant or unlabeled probes) or anti-C/EBPα. The specific DNA-protein complex bands were indicated by arrows. The sequences of various probes are shown under the panel. (D, E and F) ChIP assay to analyse C/EBPα binding to the *ACOX1* promoter in MDBK cells. DNA isolated from immunoprecipitated materials was amplified using qRT-PCR. Total chromatin was used as the input. Normal rabbit IgG was used as the negative control. A full colour version of this figure is available at https://doi.org/10.1530/JME-20-0250.

To further determine the location of C/EBPα binding sites on the *ACOX1* promoter region, EMSA and ChIP were performed, respectively. EMSA was performed with nuclear proteins extracts from bovine longissimus dorsi muscle, as shown in Fig. 5A, incubation of nuclear extracts with bio-probe1 led to the formation of a DNA-protein complex (lane 2). The quantity of the complex was decreased when cold probe was included in the reaction mixture (lane 3) but the complex formed in the presence of mutant cold probe (lane 4). Although, the DNA-protein-antibody complex was not formed after the anti-C/EBPα was added, the quantity of the DNA-protein complex was decreased (lane 5). This may be that the DNA-protein-antibody complex was too large to enter the gel. For the second and third binding sites, the DNA-protein complex was not increased in the mutant cold probe group (lane 4) compared with cold probe group (lane 3), while the quantity of the DNA–protein complex was decreased after the anti-C/EBPα was added (Fig. 5B and C). These results suggested that all of the three binding sites can bind to C/EBPα transcription factor* in vitro*. Meanwhile, these results further indicated that the second and third sites can also bind to other transcription factors.

ChIP analysis was performed in MDBK cells to determine whether C/EBPα can bind to the *ACOX1* promoter* in vivo*. Chromatin was immunoprecipitated with C/EBPα antibody and DNA fragments of the expected size were used as a template for PCR amplification. qRT-PCR was performed using primers specific to the C/EBPα binding sites in the *ACOX1* promoter (Supplementary Table 5). Compared with IgG group, all of the C/EBPα1 to 3 groups expression increased significantly (Fig. 5D, E and  F). These results confirmed that all of the three binding sites can bind to C/EBPα transcription factor *in vivo*.

### miR-25-3p directly targets *ACOX1* 3’UTR

**Figure 6:**
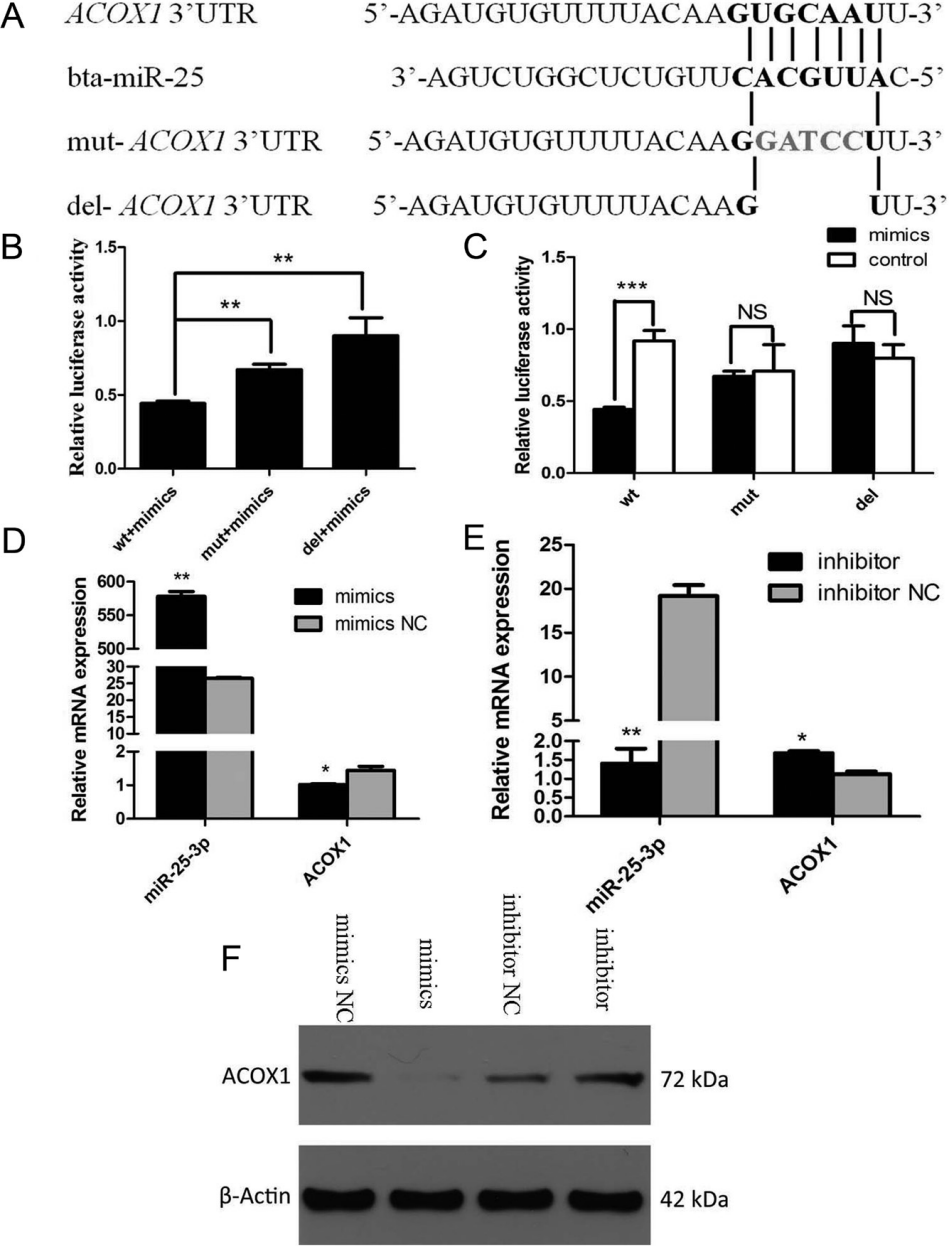
miR-25-3p directly targets the 3’UTR of ACOX1. (A) Sit-directed mutation and deletion of miR-25-3p target site in the ACOX1 3’UTR. (B and C) Dual luciferase reporter assay. ACOX1 3’UTR, mutant plasmid and deletion plasmid were transfected or co-transfected with miR-25-3p mimics/NC, respectively, into MDBK cells, dual luciferase activities were measured from cell lysates (24 h after transfection). miR-25-3p mimics/NC or inhibitor/NC were, respectively, transfected into MDBK cells. After 48 h, ACOX1 and miR-25-3p mRNA expression were detected by qRT-PCR (D, E) and ACOX1 protein expression was detected by Western blotting (F). NC, negative control (miR-239b-5p of *Caenorhabditis elegans*). β-actin served as the loading control. Data were presented as means ± s.d. (*n* ≥ 3), *P < 0.05; **P < 0.01; ***P < 0.001; NS, not significant.

To explore the post-transcriptional regulatory mechanisms of the bovine* ACOX1* gene, the possible miRNA targets were predicted using TargetScan, and a putative binding site for miR-25-3p was predicted in the 3’UTR of *ACOX1* mRNA. To validate whether miR-25-3p directly targets *ACOX1*, a luciferase reporter containing a 219 bp fragment from the *ACOX1* 3’UTR was tested* in vitro*. Additionally, we generated a mutated and a deleted version of the above mentioned reporter, in which five nucleotides of the predicted binding site were changed or deleted in order to abolish the putative interaction between miR-25-3p and *ACOX1* mRNA (Fig. 6A). The *ACOX1* 3’UTR, mutant and deletion luciferase plasmids were cotransfected with miR-25-3p mimics or NC into MDBK cells. 24 h after transfection, analyses of luciferase activity revealed that miR-25-3p mimics significantly decreased the luciferase activity of the wild reporter plasmid as compared with mutant and deletion plasmids (Fig. 6B). Meanwhile, miR-25-3p mimics significantly decreased the luciferase activity of the wild reporter plasmid as compared with NC, while there was no significant effect on the mutant and deletion plasmids (Fig. 6C). These results revealed that miR-25-3p directly targets the 3’UTR of *ACOX1 in vitro*.

To directly test the validity of the putative target, we transfected miR-25-3p mimics and miR-25-3p inhibitors into MDBK cells. The results showed that over-expression of miR-25-3p repressed *ACOX1* expression, as measured by qRT-PCR (*P*  < 0.01) (Fig. 6D) and Western blotting (Fig. 6F), whereas the knockdown of miR-25-3p derepressed it (Fig. 6E and F). These results demonstrate that the post-transcriptional activity of bovine *ACOX1* was down-regulated by miR-25-3p.

## Discussion

Intramuscular fat is indicated by the appearance of white flecks or streaks of adipose tissue between bundles of muscle fibers in skeletal muscle (Harper & Pethick 2004). Intramuscular fat content is one of the main factors for meat quality grades affecting tenderness, flavor, and juiciness of meat and plays an important role in the animal production industry (Lee *et al.* 2007, Hudson *et al.* 2015). Compared to other fatty depots, bovine intramuscular fat contains higher levels of polyunsaturated and monounsaturated fatty acids (Troy *et al.* 2016), so it has higher nutritional value. For this reason, we aimed to investigate the association of *ACOX1* gene with intramuscular adipogenesis in this study.

The accumulation of intramuscular fat is a dynamic process depending on lipogenesis, lipolysis, adipogenesis, and apoptosis. The disruption of one the these steps deeply affects intramuscular turnover. Acyl-Coenzyme A oxidase 1 (ACOX1) is the first and rate-limiting enzyme in peroxisomal fatty acid β-oxidation of fatty acids. Previous studies have found that *ACOX1* was correlated with the meat quality of livestock (Casas Carrillo *et al.* 1997, Clop *et al.* 2003, Yue *et al.* 2003, Lee *et al.* 2010, Jiao *et al.* 2011), while the role of *ACOX1* in intramuscular adipogenesis of beef cattle was not clear. In the present study, we performed gain-of-function and loss-of-function experiments in bovine intramuscular preadipocytes to investigate whether *ACOX1* was a regulator of intramuscular adipogenesis. Taken together, the data showed that *ACOX1* promoted lipid accumulation of bovine intramuscular preadipocytes. The level of triglyceride was increased by *ACOX1*, while levels of ATP and ROS were reduced. Meanwhile, Western blotting results showed that the expressions of adipogenic differentiation gene (*C/EBPβ*) and fatty acid synthesis genes (*FAS* and *ACC*) were induced by *ACOX1*, while lipolysis genes (*LPL* and *HSL*) expressions were inhibited. These data indicated that *ACOX1* promotes adipogenesis of bovine intramuscular preadipocytes in terms of phenotype, gene expression and cell contents.

To further understand the transcriptional regulatory mechanism of *ACOX1*, we analyzed the 5’-flanking region of bovine *ACOX1* gene via AliBaba2.1 and MatInspector. Bioinformatic analysis revealed that there were three potential C/EBPα transcription factor binding sites, located at −1142 to −1129 bp, −831 to −826 bp, and −303 to −298 bp, respectively. Thus, five fragments of 5’-flanking sequences of bovine *ACOX1* gene were isolated. Subsequently, a series of experiments, including dual luciferase, site-directed mutagenesis, EMSA, ChIP and qRT-PCR assays, confirmed that C/EBPα suppressed transcription of bovine *ACOX1* gene via binding to three C/EBPα binding sites in the *ACOX1* promoter.

CCAAT-enhancer binding protein (C/EBP), a member of the basic leucine zipper (bZIP) transcription factor family, is named for its ability to bind to CCAAT sequences on many gene promoters (Landschulz *et al.* 1988). It is a family of transcription factors that include C/EBPα, C/EBPγ, C/EBPβ, C/EBPCδ, C/EBPε and C/EBPζ (Li *et al.* 2004). Among them, C/EBPα, C/EBPβ and C/EBPCδ are involved in regulating adipocyte differentiation. In addition to PPARγ, C/EBPα is the most important factor for regulating lipid deposition and adipocyte differentiation, and its key role is mainly manifested in the terminal differentiation stage of adipocytes. During terminal differentiation, C/EBPα is induced by C/EBPβ and C/EBPCδ, and once expressed, it is activated and maintained by its own C/EBP effector domain (Christy *et al.* 1991).

Numerous studies have demonstrated that many fat-specific genes promoter have C/EBP effector domain that can be activated by C/EBPα (Macdougald & Lane 1995). For instance, C/EBPα could induce *PPARγ* expression by identifying the C/EBP effector domain of the *PPARγ* promoter (Wu *et al.* 1999), and C/EBPα may act as a positive regulator binding to fat mass and obesity associated gene (*FTO*) promoter and activates the gene transcription (Ren *et al.* 2014). Meanwhile, previous studies have suggested that C/EBPα, acted as the transcription factor, could regulate many genes expression. For example, C/EBPα regulates transcription of human fructose-1,6-bisphosphatase (*FBP1*) gene via binding to the two overlapping C/EBPα binding sites located at nucleotide -228/-208 (Wattanavanitchakorn *et al.* 2018), C/EBPα binding to the human polo-like kinase 1 (*PLK1*) promoter results in suppressed *PLK1* expression (Dasgupta *et al.* 2017). Furthermore, there were two C/EBPα binding sites in the chicken cytochrome P450 (CYP) 2D49 promoter, and over-expression of C/EBPα significantly upregulated *CYP2D49* transcription (Yang *et al.* 2014). In this study, we identified that C/EBPα binds to the *ACOX1* promoter region and suppressed its transcription activity.

miRNAs are endogenous, small (~22 nucleotides), and single-stranded noncoding RNAs. The role of different miRNAs in biological systems is well established. They are generally regarded as negative regulators of gene expression, as they bind to the 3’UTR of messengerRNAs (mRNAs), leading to mRNA degradation and/or suppression of mRNA translation (Bartel 2004, Carthew & Sontheimer 2009, Malan-Mueller *et al.* 2013). Previously, we have reported that miR-25-3p could reduce the level of triglyceride and increased the levels of ATP and ROS, this was exactly contrary to what *ACOX1* does (Zhang *et al.* 2018). Therefore, we speculated that *ACOX1* might be regulated by miR-25-3p. First, we searched for potential miRNAs of bovine *ACOX1* gene via TargetScan. Fortunately, the 3’UTR of *ACOX1* contained a seven nucleotides perfect match site complementary to the miR-25-3p seed region (Fig. 6A). Then, the dual luciferase reporter assay demonstrated that *ACOX1* was a direct target of miR-25-3p, shown by the steady decrease luciferase activity of the wt vector; but not the mutant and deletion form (Fig. 6B and C). Meanwhile, qRT-PCR and Western blotting results showed that the expression of *ACOX1* was inhibited by the miR-25-3p mimics, and that this inhibition was reversed by the miR-25-3p inhibitors (Fig. 6D, E and F). These results suggested that the post-transcriptional activity of *ACOX1* was suppressed by miR-25-3p.

In conclusion, our results demonstrate that *ACOX1* gene acts as a positive regulator of the adipogenesis of bovine intramuscular preadipocytes. Moreover, the transcriptional and post-transcriptional activity of *ACOX1* was regulated by C/EBPα and miR-25-3p, respectively.

## Supplementary Material

Figure S1.The mRNA expression of ACOX1 was detected by qRT-PCR. Data were presented as means ± SD (n = 3), *P < 0.05.

Figure S2.The mRNA expression of C/EBPα was detected by qRT-PCR. Data were presented as means ± SD (n = 3), *P < 0.05.

Table S1 Primers for qRT-PCR

Table S2 Primers for amplification of bovine ACOX1 promoters and C/EBPα transcription factor binding sites mutated fragment

Table S3 Primers for amplification of ACOX1 3' UTR and its mutated and deleted fragment

Table S4 Primers for EMSA assays

Table S5 Primers for ChIP assays

## Declaration of interest

The authors declare that there is no conflict of interest that could be perceived as prejudicing the impartiality of the research reported.

## Funding

This research was supported by the Science Foundation Project of Hubei Academy of Agricultural Sciences (2019NKYJJ04), China Postdoctoral Science Foundation (2017M610465), National Key Research Development Program of China (2017YFD0502000), Hubei Key Projects of Technical Innovation (2019ABA084) and Hubei Agricultural Science and Technology Innovation Action Project (2018skjcx05).
