# Supplementary figures and images for: ACOX1, regulated by C/EBPα and miR-25-3p, promotes bovine preadipocyte adipogenesis
Source: J Mol Endocrinol. 2021 Jan 22;66(3):195–205. doi: 10.1530/JME-20-0250 (PMC8052523; doi:10.1530/JME-20-0250)

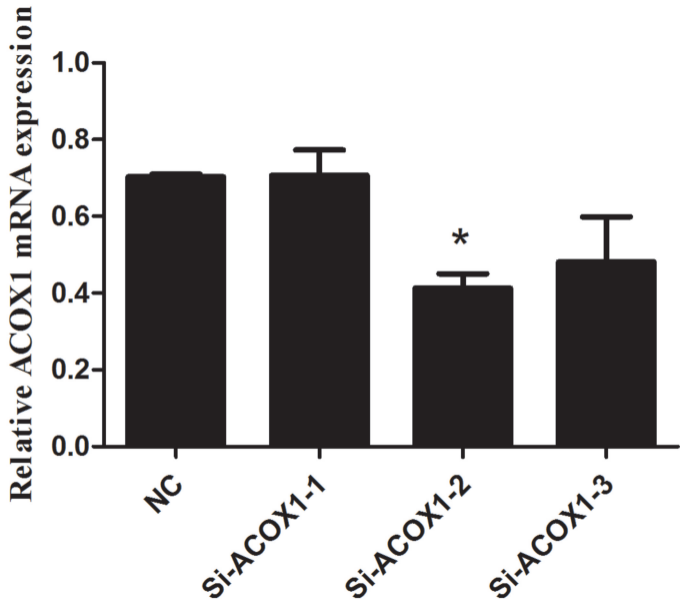

Supplement: Figure S1.The mRNA expression of ACOX1 was detected by qRT-PCR. Data were presented as means ± SD (n = 3), *P < 0.05. [file supplementary_figure_1.pdf]

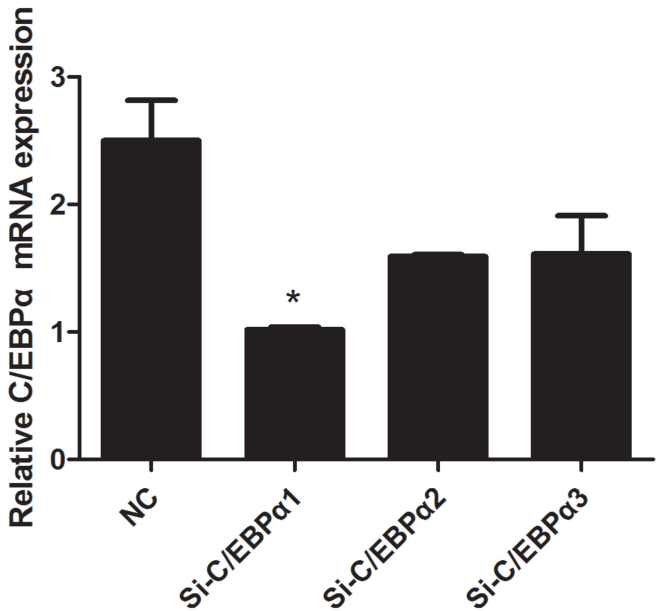

Supplement: Figure S2.The mRNA expression of C/EBPα was detected by qRT-PCR. Data were presented as means ± SD (n = 3), *P < 0.05. [file supplementary_figure_2.pdf]
