# Supplementary material for: ACOX1, regulated by C/EBPα and miR-25-3p, promotes bovine preadipocyte adipogenesis
Source: J Mol Endocrinol. 2021 Jan 22;66(3):195–205. doi: 10.1530/JME-20-0250 (PMC8052523; doi:10.1530/JME-20-0250)
Supplement: Table S1 Primers for qRT-PCR [file supplementary_table_1.pdf]

Table S1 Primers for qRT-PCR

Primer	Primer sequence（5'-3'）	Size(bp)	Tm (℃)
miR-25-3p RT	CTCAACTGGTGTCGTGGAGTCGGCAATTC<br>AGTTGAGTCAGACCG		
miR-25-3p	F: CTGGTAGGCATTGCACTTGTCT<br>R: TCAACTGGTGTCGTGGAGTCGGC		60
U6	F: GCTTCGGCAGCACATATACTAAAAT<br>R(RT): CGCTTCACGAATTTGCGTGTCAT		60
ACOX1	F: GTGGATATCAACAGCCCCGA<br>R: GAATCTGGAGGACTTTTTCCGT	217	58
C/EBPα	F: CAAAGCCAAGAAGTCCG<br>R: GCTCAGTTGTTCCACCC	181	58
β-actin	F: TTAGCTGCGTTACACCCTT<br>R: TGTCACCTTCACCGTTCC	169	58
