# Supplementary material for: ACOX1, regulated by C/EBPα and miR-25-3p, promotes bovine preadipocyte adipogenesis
Source: J Mol Endocrinol. 2021 Jan 22;66(3):195–205. doi: 10.1530/JME-20-0250 (PMC8052523; doi:10.1530/JME-20-0250)
Supplement: Table S2 Primers for amplification of bovine ACOX1 promoters and C/EBPα transcription factor binding sites mutated fragment [file supplementary_table_2.pdf]

Table S2 Primers for amplification of bovine *ACOX1* promoters and C/EBPα transcription factor binding sites mutated fragment

Name	Primer sequence (5'-3')	Size (bp)	Tm (℃)
ACOX1-P1	F: **GG**<u>GGTACC</u>GCAGAGACATCACTTTGTCA<br>R: **CCG**<u>CTCGAG</u>GACAGCAGGAATCCGCAGCT	1235	60
ACOX1-P2	F: **GG**<u>GGTACC</u>ACTGATGCTGAAGCTGAAGC<br>R: **CCG**<u>CTCGAG</u>GACAGCAGGAATCCGCAGCT	1012	60
ACOX1-P3	F: **GG**<u>GGTACC</u>TATCTCACGCTCTGCTTCCC<br>R: **CCG**<u>CTCGAG</u>GACAGCAGGAATCCGCAGCT	714	60
ACOX1-P4	F: **GG**<u>GGTACC</u>CGAGCCATCTCAAGAAGGCC<br>R: **CCG**<u>CTCGAG</u>GACAGCAGGAATCCGCAGCT	551	60
ACOX1-P5	F: **GG**<u>GGTACC</u>CAATTCCTTGACCTTCTTCC<br>R: **CCG**<u>CTCGAG</u>GACAGCAGGAATCCGCAGCT	263	60
Mut1	F: TTTGAACTGTGGTGggatAGAAGCCTCTTGAG<br>R: CTCAAGAGGCTTCTatccCACCACAGTTCAAA		
Mut2	F: GCAGTCCATGGGGTtatcAGGAGTCGGACAGG<br>R: CCTGTCCGACTCCTgataACCCCATGGACTGC		
Mut3	F: ACCACCACCACCTGaatcATTCCTTGACCTTC<br>R: GAAGGTCAAGGAATgattCAGGTGGTGGTGGT		

Note: underlined, enzyme loci; bold, protective bases, lowercase, mutations base
