# Supplementary material for: ACOX1, regulated by C/EBPα and miR-25-3p, promotes bovine preadipocyte adipogenesis
Source: J Mol Endocrinol. 2021 Jan 22;66(3):195–205. doi: 10.1530/JME-20-0250 (PMC8052523; doi:10.1530/JME-20-0250)
Supplement: Table S3 Primers for amplification of ACOX1 3' UTR and its mutated and deleted fragment [file supplementary_table_3.pdf]

Table S3 Primers for amplification of *ACOX1* 3' UTR and its mutated and deleted fragment

| Name | Primer sequence（5' - 3'） |
|---|---|
| ACOX1-3’UTR-F | **GG**GTTTAAACACTTCGTGGAATCTTG |
| ACOX1-3’UTR-R | **CCG**CTCGAGGGATCAGCAGCAATAT |
| Mut-F | ATGTGTTTTACAAGgatccTTAACACTGAAAGA |
| Mut-R | TCTTTCAGTGTTAAggatcCTTGTAAAACACAT |
| Del-F | ATGTGTTTTACAAGTTAACACTGAAAGA |
| Del-R | TCTTTCAGTGTTAACTTGTAAAACACAT |

**Note**: underlined, enzyme loci; bold, protective bases, lowercase, mutations bases
