# Supplementary material for: ACOX1, regulated by C/EBPα and miR-25-3p, promotes bovine preadipocyte adipogenesis
Source: J Mol Endocrinol. 2021 Jan 22;66(3):195–205. doi: 10.1530/JME-20-0250 (PMC8052523; doi:10.1530/JME-20-0250)
Supplement: Table S4 Primers for EMSA assays [file supplementary_table_4.pdf]

Table S4 Primers for EMSA assays

Name	Primer sequence（5' - 3'）
Bio1-F	bio-ACTGTGGTGTTGCAGAAGCCTC-bio
Bio1-R	bio-GAGGCTTCTGCAACACCACAGT-bio
wt1-F	ACTGTGGTGTTGCAGAAGCCTC
wt1-R	GAGGCTTCTGCAACACCACAGT
Mut1-F	ACTGTGGTGggatAGAAGCCTC
Mut1-R	GAGGCTTCTatccCACCACAGT
Bio2-F	bio-CCATGGGGTCGCAAGGAGTCGG-bio
Bio2-R	bio-CCGACTCCTTGCGACCCCATGG-bio
wt2-F	CCATGGGGTCGCAAGGAGTCGG
wt2-R	CCGACTCCTTGCGACCCCATGG
Mut2-F	CCATGGGGTtatcAGGAGTCGG
Mut2-R	CCGACTCCTgataACCCCATGG
Bio3-F	bio-CACCACCTGGGCAATTCCTTGA-bio
Bio3-R	bio-TCAAGGAATTGCCCAGGTGGTG-bio
wt3-F	CACCACCTGGGCAATTCCTTGA
wt3-R	TCAAGGAATTGCCCAGGTGGTG
Mut3-F	CACCACCTGaatcATTCCTTGA
Mut3-R	TCAAGGAATgattCAGGTGGTG

Note: Red, core binding sites; lowercase, mutations base
