# Supplementary material for: ACOX1, regulated by C/EBPα and miR-25-3p, promotes bovine preadipocyte adipogenesis
Source: J Mol Endocrinol. 2021 Jan 22;66(3):195–205. doi: 10.1530/JME-20-0250 (PMC8052523; doi:10.1530/JME-20-0250)
Supplement: Table S5 Primers for ChIP assays [file supplementary_table_5.pdf]

Table S5 Primers for ChIP assays

Name	Primer sequence (5’-3’)	Size (bp)	Tm (℃)
ChIP- C/EBPα1	F: AGAGTTGGACTATAAAGAA<br>R: GCATCAGTCCTTCCA	156	55
ChIP- C/EBPα2	F: GGTGATGGACAGGGAAGG<br>R: CCGCATTTATTATGGGTTG	109	55
ChIP- C/EBPα3	F: CCGCACCACCACCACCACCT<br>R: CGGGCGAGTCCCCTGTTCT	105	65
